# Diurnal activity in cane toads (*Rhinella marina*) is geographically widespread

**DOI:** 10.1038/s41598-020-62402-3

**Published:** 2020-03-31

**Authors:** Lachlan Pettit, Simon Ducatez, Jayna L. DeVore, Georgia Ward-Fear, Richard Shine

**Affiliations:** 1 School of Life and Environmental Sciences, University of Sydney, Sydney, 2006 Australia; 2 Centre de Recerca Ecologica i Aplicacions Forestals - CREAF, Universitat Autonoma de Barcelona, Cerdanyola del Vallès, Catalunya 08193 Spain; 3 Department of Biological Sciences, Macquarie University, Sydney, 2019 Australia

**Keywords:** Behavioural ecology, Invasive species

## Abstract

Although adult cane toads (*Rhinella marina*) are generally active only at night, a recent study reported that individuals of this species switched to diurnal activity in response to encountering a novel habitat type (deeply shaded gorges) in the course of their Australian invasion. Our sampling over a broader geographic scale challenges the idea that this behaviour is novel; we documented diurnal behaviour both in the species’ native range and in several sites within the invaded range, in multiple habitat types. Diurnal activity was most common in the tropics and in areas where toads attain high population densities and are in poor body condition, suggesting that the expansion of activity times may be induced by intraspecific competition for food.

## Introduction

All animals are active for only a limited period of the diel cycle, and that timing is so consistent that we commonly define species as nocturnal (e.g., owls), diurnal (e.g., bearded dragons) or crepuscular (twilight hours; e.g., microbats). Organisms have evolved these life history traits to cope with the physiological demands of activity^1^; to exploit different niches^2^; and to temper various ecological interactions (such as predation^3^). Nonetheless, facultative shifts in the diel timing of activity are common even within a species. For example, animals that are usually nocturnal may be driven to diurnal behaviour by factors such as nutritional stress, thermal extremes, predation risk and intraspecific competition^4^.

In many species of anuran amphibians (frogs and toads), adults are normally active only at night^5^. Recently, Doody *et al*.^6^ described a switch to diurnal behaviour in a species (the cane toad, *Rhinella marina*) that is usually nocturnal. Camera-trapping revealed that toads were nocturnally active in nearby sun-exposed gorges but were often active by day in shaded gorges. The authors attributed that shift as a response to novel conditions (deep shade) that do not occur elsewhere in the toads’ broad distribution. In the course of our own fieldwork, we have sampled over a greater geographic range (but for shorter times at each place) and find that diurnal activity occurs in multiple populations of cane toads, in widely separated areas, and in several habitat types. Our radiotracking of cane toads in French Guiana revealed diurnal activity in native-range populations as well.

Our data challenge Doody *et al*.’s^6^ interpretation that diurnal activity of toads at their study sites is a novel response, elicited by a novel habitat type^6^. Why, then, are toads sometimes active by day? Plausible explanations include diurnality as a response to:

1. availability of cool moist conditions by day, reducing the abiotic challenges of diurnal foraging;
2. compensation for restricted availability of sufficiently high temperatures at night, allowing foraging to take place over a longer period of time;
3. food scarcity, such that animals need to forage over a longer period in order to meet their energy requirements.

The first two of these hypotheses predict that diurnal behaviour should be most common in cooler and moister parts of the toads’ geographic range, whereas the third hypothesis predicts the opposite pattern (i.e., we expect more diurnality in hotter regions, because high temperatures increase metabolic rates and thus maintenance requirements) and also, predicts that diurnality will occur in areas where toads are abundant (increasing intraspecific competition for limited prey resources) and are in poor body condition. To test these competing predictions, we regressed the incidence of diurnal behaviour against other site-specific parameters.

## Methods

### Standardised surveys in australia

We quantified the abundance of cane toads by day and by night at sites along two continent-scale transects, each covering the full 84-year timespan of the toad invasion chronosequence in Australia (Table 1). The “east coast transect” (surveyed October 2017 to April 2018) consists of 16 sites running north-south, beginning near Townsville (close to the original release locations in 1935) and extending down the east coast of Australia to the southern invasion front in northern New South Wales. We focused our surveys at campsites in national parks and reserves and the surrounding temperate woodland. We also surveyed 18 sites along the toads’ trajectory of invasion east-west across the wet/dry tropics (the tropical transect, January to May 2019) between Townsville (as above) and Oombulgurri (recently invaded) in Western Australia. Each tropical site bordered a riparian area (river, dam, etc) and consisted of floodplain and tropical woodland savannah habitats.

**Table 1:** The location of study sites in Australia along transects where cane toads (*Rhinella marina*) have invaded across tropical Australia’s wet/dry tropics (n = 18) and through temperate woodlands along the east coast of Australia (n = 16). Four sites from toads’ native range in French Guiana are included, as well.

Transect	Site	State/Region	Years since invasion	Annual rainfall (mm)	Latitiude	Longitude	Habitat feature	Broad vegetation community
Northern	Allison River	Western Australia	6	831.9	−15.144051	127.851948	River	Savanna floodplain
Northern	Bowling Green Bay	Queensland	84	1128	−19.315085	147.023585	Wetland	Coastal floodplain
Northern	Buffalo Farm	Northern Territory	15	1564.9	−12.815603	132.594466	Billabong	Savanna floodplain
Northern	Buttons Crossing	Western Australia	9	841.3	−15.620739	128.692428	River	Savanna floodplain
Northern	Casuarina Reserve	Northern Territory	13	1731.2	−12.353451	130.872674	Tidal creek	Tropical woodland
Northern	Copperfield River	Queensland	39	738.2	−19.467397	144.157654	River	Eucalypt woodland
Northern	Darram Conservation Reserve	Western Australia	9	841.3	−15.7991	128.690715	River	Savanna floodplain
Northern	East Point Reserve	Northern Territory	13	1731.2	−12.411873	130.821	Tidal creek	Tropical woodland
Northern	Emu Swamp	Queensland	39	738.2	−19.416468	144.162622	Dam	Eucalypt woodland
Northern	Fogg Dam	Northern Territory	14	1393.9	−12.558321	131.296231	Dam	Savanna floodplain
Northern	Goose Hill Creek	Western Australia	8	831.9	−15.57077	128.356428	River	Savanna floodplain
Northern	Harrison Dam	Northern Territory	14	1393.9	−12.560388	131.340188	Dam	Tropical savanna
Northern	Lawn Hill	Queensland	33	613.6	−18.701113	138.492113	River	Gallery rainforest
Northern	Miyumba	Queensland	33	613.6	−19.020244	138.718685	River	Gallery rainforest
Northern	Nourlangie Camp	Northern Territory	15	1564.9	−12.761612	132.663956	River	Savanna floodplain
Northern	Oombulgurri Swamp	Western Australia	6	831.9	−15.181047	127.843459	Swamp	Savanna floodplain
Northern	Parrys Lagoon	Western Australia	8	831.9	−15.549525	128.258414	Lagoon	Savanna floodplain
Northern	Town Common	Queensland	84	1128	−19.200396	146.755701	Wetland	Coastal floodplain
East Coast	Alligator Creek	Queensland	80	1128	−19.43428	146.946228	Wetland	Eucalypt woodlands to open forest
East Coast	Bar Mtn Access	New South Wales	1	1457.2	−28.500195	153.117355	Campground	Sub-tropical & warm temperate rainforest
East Coast	Broken River	Queensland	72	2199	−21.16905	148.506119	Campground	Rainforest and scrub
East Coast	Bymien	Queensland	62	1468.6	−25.954248	153.103973	Picnic ground	Rainforest and scrub
East Coast	Cocoa Creek	Queensland	76	1128	−19.291119	147.004013	Campground	Eucalypt woodlands to open forest
East Coast	Cutters Camp	New South Wales	8	1457.2	−28.445637	153.194382	Campground	Sub-tropical & warm temperate rainforest
East Coast	Eurimbula Creek	Queensland	42	1162.8	−24.173948	151.849442	Campground	Eucalypt woodlands to open forest
East Coast	Freshwater	Queensland	62	1468.6	−26.000885	153.147385	Campground	Eucalypt woodlands to open forest
East Coast	Harrys Hut	Queensland	62	1468.6	−26.189236	153.029709	Campground	Melaleuca open woodlands
East Coast	Korrumbyn	New South Wales	28	1395	−28.392597	153.300903	Picnic ground	Sub-tropical & warm temperate rainforest
East Coast	Middle Creek	Queensland	42	1162.8	−24.126186	151.783264	Campground	Eucalypt woodlands to open forest
East Coast	Redwood	Queensland	43	820.8	−27.563951	151.997452	Picnic ground	Eucalypt woodlands to open forest
East Coast	Sheepstation Creek	New South Wales	5	1632.2	−28.413572	153.023041	Campground	Sub-tropical & warm temperate rainforest
East Coast	Smalleys Beach	Queensland	69	1603	−20.913721	149.017059	Picnic ground	Eucalypt woodlands to open forest
East Coast	Woombah	New South Wales	13	1306.3	−29.359802	153.28215	Picnic ground	Northern open grassy Blackbutt
East Coast	Wreck Rock	Queensland	42	1162.8	−24.316492	151.963104	Campground	Eucalypt woodlands to open forest
French Guiana	Gosselin	Rémire-Montjoly	Native range	2815.8	4.890805556	−52.2530556	Freshwater seeps	Beach bordered by rainforest fragments
French Guiana	Kaw Fourgassier	Roura	Native range	3364.6	4.643666667	−52.2991167	Pond	Rainforest
French Guiana	Montjoly	Rémire-Montjoly	Native range	2815.8	4.913283333	−52.2598667	Freshwater rock pools	Beach bordered by rainforest fragments
French Guiana	Regina Wash	Regina	Native range	3364.6	4.363283333	−52.2798667	Stream	Rainforest

Each site was surveyed over two sessions for a total of five days, with each survey session lasting two or three days. For logistical reasons up to three sites were surveyed concurrently, in randomised order to avoid latitudinal, longitudinal and seasonal bias. We also randomised the order that grouped sites were surveyed each day to remove time-of-day bias. We combined active search surveys and baited remote-sensing camera stations to estimate the number of cane toads and determine their times of activity.

#### Active search surveys

All toads encountered during surveys were recorded on mobile application software (Sightings v1.01). Survey effort was standardised (1 h/survey). Diurnal surveys were conducted on sunny days with maximum air temperature above 23 °C, and nocturnal surveys were conducted on dry nights with temperatures above 17 °C. Survey protocols differed slightly between transects due to targeting different varanid lizards in a concurrent project. The east coast transect sites were surveyed three times per day: morning (0800–1200 EST), afternoon (1200–1845 EST) and night (1845–0030). Each survey was partitioned into a 15-minute active-search on foot around target campsites, and a 45-minute active-search along a 5 km transect by vehicle through surrounding bushland. The tropical transect sites also were surveyed three times per day: morning (beginning 30 min after sunrise), afternoon (commencing three hours before sunset) and night (beginning 30 min after sunset). One-hour morning active search surveys were conducted on foot along a two km transect near focal waterbodies (rivers, creeks, dams, lagoons and billabongs). The afternoon and night surveys both involved a 30-min active search survey on foot (~1 km) and 30-min active survey along a five-km transect by vehicle (car or quad bike).

We sampled a subset of toads during each nocturnal survey. We sexed, weighed (g), and measured snout-urostyle length (“SUL”, to nearest 0.1 mm) of 962 adult toads, then gave an identifying toe clip prior to release at their point of capture. To avoid pseudoreplication, we excluded all recaptures from analysis. Body condition was calculated as a scaled mass index using the formula M_i_ * (L_0_/L_i_)^b^SMA^, with Mi and L_i_ as the mass and length of the individual, L_0_ as the mean body length, and b^SMA^ as the slope of the sex specific standard major axis log-log regression of mass by SUL for measured adults^7^.

#### Remote camera surveys

We deployed eight remote-sensing cameras (Scoutguard SG560K) and bait stations at each site. Cameras at east coast sites were positioned in two 100 m grids, one surrounding focal campsites and the other in bushland two km away, and were deployed for 48 hours (total 16 trap days/nights per site). Cameras at tropical transect sites were positioned near waterbodies along the active search survey transect, spaced at least 100 m apart, and deployed for two sessions lasting 48 and 72 h (total 40 trap days/nights per site).

Cameras were positioned on trees at a height of 40 cm, oriented towards the south, and placed in areas shaded by canopy cover where possible. A bait containing one chicken neck (east coast transect) or 80 g of sardines in oil (tropical transect) was placed one m from the camera in a PVC cannister attached to star picket at a height of 30 cm (such that it was non-consumable by vertebrate predators). Additional consumable baits were added around the base of most bait stations. Most sites (10/16) along the east coast transect had a cracked chicken egg placed on the ground at half of the bait stations (for a concurrently-run behavioural experiment). All bait stations deployed at tropical transect sites had one chicken egg (with small crack in the shell to release olfactory cues) placed at the base of the picket. A sardine and a cane toad leg (collected from road-killed cane toads and washed in water, frozen, and thawed 2 hours prior to deployment) were placed 30 cm to either side of the picket and covered with a plastic lid with mesh window (20 × 27 cm), with the position of sardine and toad leg randomised. Video footage showed toads feeding on the invertebrates that were attracted to both consumable and non-consumable baits.

Each camera was set to record one minute of video when triggered. Many videos contained images of more than one toad, and given the video resolution, we could not confidently identify individual toads across multiple videos. Our abundance estimates used a 30-minute event period to determine the number of active toads. When an animal was first detected on video, we reviewed videos from the next 30 minutes, and the video with the highest number of simultaneously visible toads within the timeframe was used as our abundance count for that period. Finally, we classified each toad as either diurnal (sighted or filmed between sunrise and sunset) or nocturnal (detected at night).

### Radiotelemetry in French Guiana

We radio-tracked 34 cane toads at four sites (two coastal beach sites and two within the Amazon rainforest) in French Guinea between Aug and Sep 2017 in order to quantify toad activity in their native range. We hand-captured 10 toads at each site (except one rainforest site at Kaw Fourgassier, where only 4 toads were found), measured and weighed them, and determined their sex based on morphology (skin rugosity, color, the presence of nuptial pads) and male-specific “release calls”. We then attached radio-transmitters (Holohil PD-2, ~3.5 g, <5% of toad mass) to cotton twine waist-belts. Toads were equipped and released at their point of capture within 15 min of capture.

We then located each animal every day (between sunrise and sunset) for five days. During that period, each toad was also located on three nights (2000 to 0100). At the end of this sampling period the radio-transmitters were removed. Three toads at one site (the beach site of Montjoly) either dropped their transmitters or moved to inaccessible private land during the survey, so we had fewer observations for these individuals. Each time a toad was located, it was scored as either “inactive” or “active”. A toad was considered inactive if it was crouched or nestled within a refuge site (e.g. under thick vegetation or within a crevice). Body condition was calculated using the same scaled mass index described above (as estimated from a dataset of 240 adult toads measured in French Guiana in Aug-Sep 2017).

## Statistical Analysis

### Standardised surveys in Australia

Our dependent variable was the number of toads scored as active by day, as a proportion of the total number of toads observed at that site (i.e., combining both camera-trap and active-survey counts). Homoscedasticity was checked with Levene’s test and normality was assessed with Shapiro Wilk-W tests for all data. When data diverged from parametric assumptions we applied an appropriate function or analysed data with a non-parametric test. All statistical analyses were performed with JMP (ver.13).

#### Correlations of diurnal behaviour with ambient conditions

We collated data on mean maximum temperature and total rainfall (Bureau of Meteorology, retrieved 18 Oct 2019) for the eight months of the year (Oct–May) when toads are most active. Because our data did not meet assumptions of normality or homoscedasticity, we used Wilcoxon rank-based tests to examine if sites where at least some toads were diurnally active were cooler or wetter than sites where all toads were nocturnal. We also collated detailed weather data at 30-minute intervals (Bureau of Meteorology, retrieved 15 Sep 2019) for the two sites with the highest proportion of diurnal activity (Oombulgurri Swamp and Townsville Town Common). Our data (raw or transformed) did not meet the assumptions of parametric tests. However, linear model results are robust when sample sizes are large^8^. Accordingly, we used linear mixed effects models fit by restricted maximum likelihood estimation to test the null hypothesis that weather conditions (temperature and relative humidity) at each site do not differ between daytime and nighttime toad sightings, with day included as a random factor. Rejecting the null hypothesis would indicate that toads active during the daytime experienced abiotic conditions different to those experienced by toads encountered during night-time activity.

#### Correlation of abundance of toads with diurnal activity

We used a Spearman test to examine if the rank proportion of toads that were diurnal in each population was correlated with the rank densities of toads.

#### Correlation of toad body condition with diurnal activity

We used a one-way analysis of variance (ANOVA) to test if the body condition scores of toads from populations that exhibited purely nocturnal activity were higher when compared with populations where we recorded diurnal as well as nocturnal activity. We included “activity” (exclusively nocturnal vs both diurnal and nocturnal) as a fixed factor, and site nested within activity to account for variation between sites. Our data had equal variances, but assumptions of normality of residuals could not be met. Due to large sample sizes our results are robust to these deviations from normality^8^.

### Radiotelemetry in French Guiana

Due to the small number of toads tracked in the native range, we only report the proportion of toads that were diurnally or nocturnally active. We used one-way ANOVAs to test if native-range toads that exhibited diurnal activity differed in size (SUL [mm], log transformed) or body condition from toads that were exclusively nocturnal.

All procedures were approved by the University of Sydney ethics committee (approval 2017/1202) and were carried out in accordance with relevant guidelines and regulations under licence from state and federal wildlife agencies. French Guiana field work was conducted under the French Ministère de la Transition Ecologique et Solidaire permit TREL1734890A/1 (19 December 2017) and the arrêté from the Préfet de la Région Guyane APmodif-R03-2017-07-18-006.

## Results

### Standardised surveys in Australia

**Figure 1:**
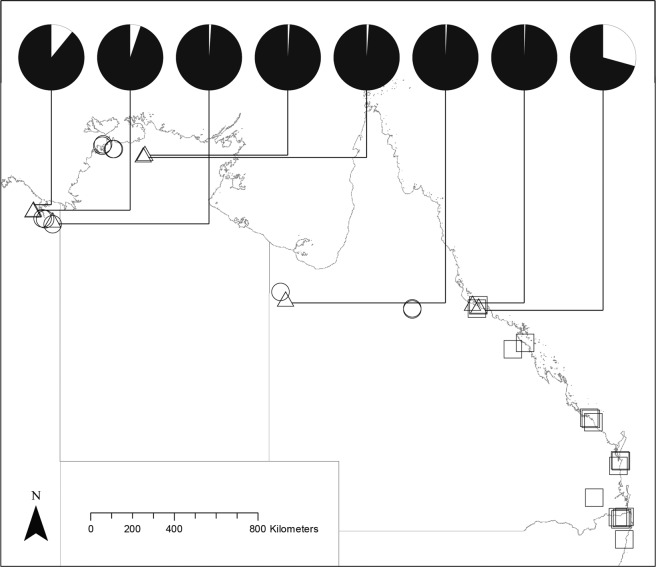
The time of day that cane toads (*Rhinella marina*) were active was determined with active search surveys and remote-sensing cameras at 34 sites across the southern and western cane toad invasion ranges. Triangles designate sites where at least some toads where diurnally active. Circles (tropical transect) and squares (east coast transect) depict sites with nocturnal activity only. Pie charts show the proportion of toads that were diurnally (white) and nocturnally (black) active at respective sites. Sites without an accompanying pie chart are those where only nocturnal activity was detected.

**Figure 2:**
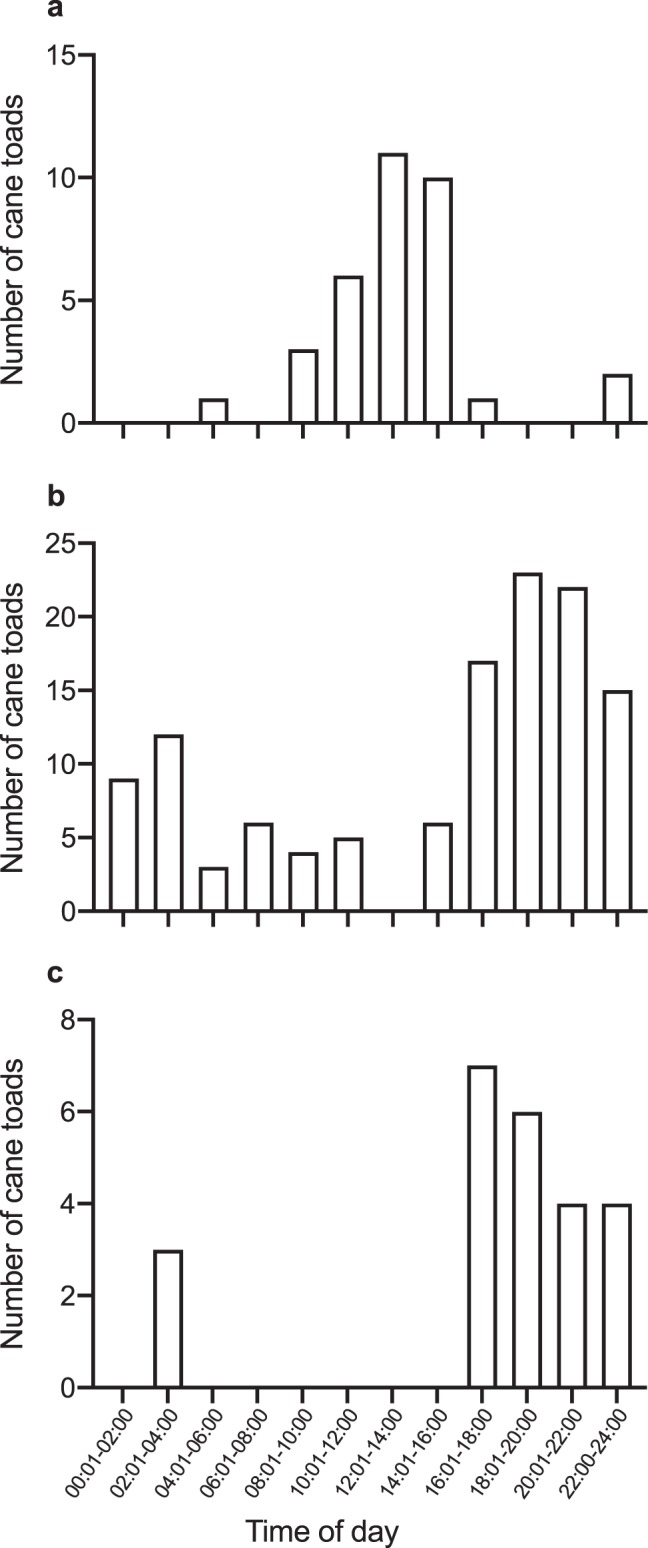
Frequency histograms showing the time of day that active cane toads (*Rhinella marina*) were detected at bait stations by remote-sensing cameras at three sites. Cane toads attracted to bait stations were primarily diurnal at (**a**) Oombulgurri Town Swamp (invaded for 6 years) and were active both by day and by night at (**b**) Townsville Town Common (invaded for 84 years), whereas toads from (**c**) Parry’s Lagoon (invaded for 8 years) were primarily nocturnal.

**Figure 3:**
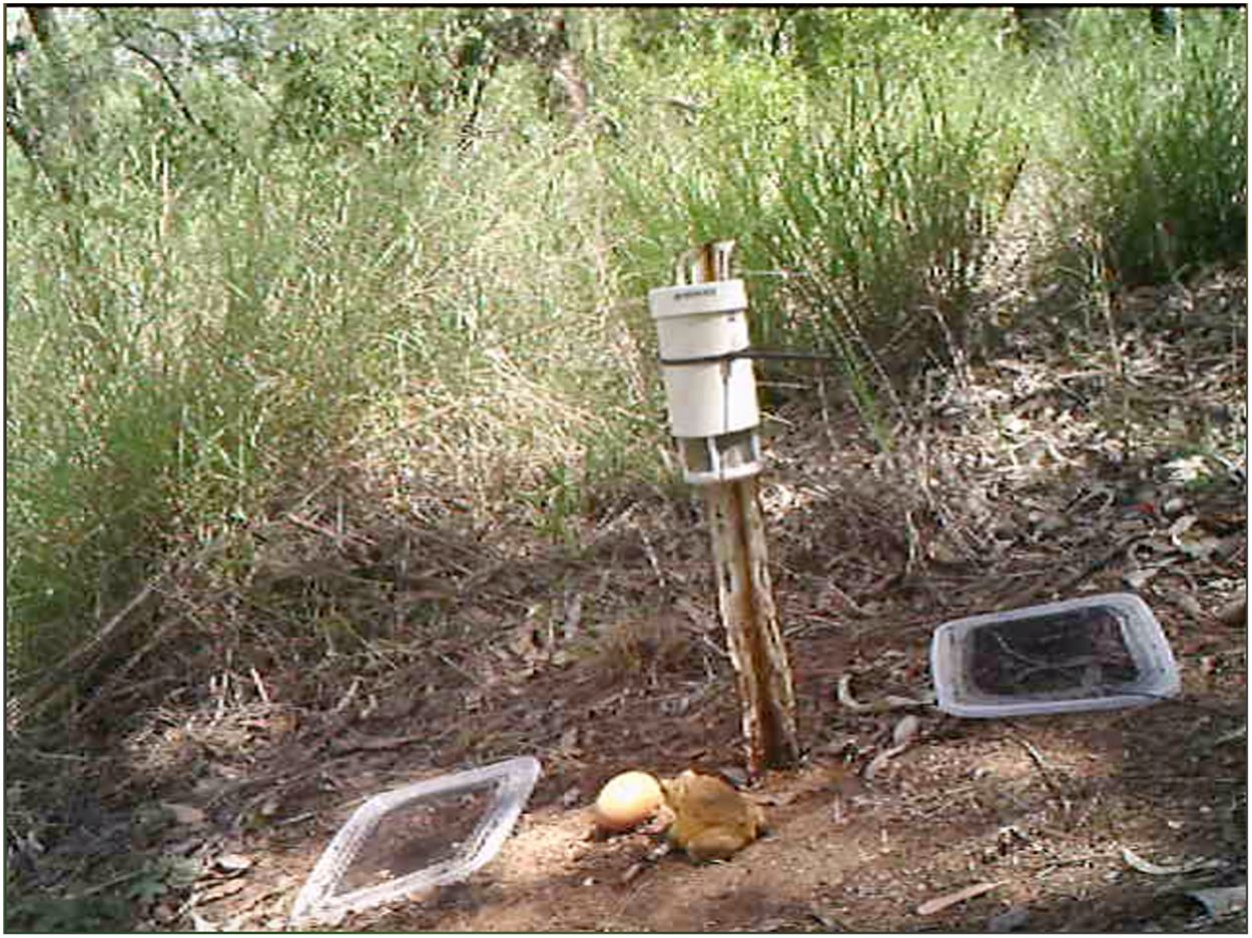
Image from a camera trap of an adult cane toad (*Rhinella marina*; in central foreground) feeding on invertebrates attracted to a bait station (a cracked chicken egg) at Miyumba (Gregory River) in Queensland. This toad foraged in an exposed location (over 40 m to nearest water body) for 34 minutes; ambient air temperature was 37.7 °C.

Our five-day surveys detected diurnally active cane toads at 44% (8/18) of sites from across the tropical transect, but not at any sites along the east coast transect (0/16). The eight sites with diurnal toads were spread across the invasion chronosequence in tropical Australia, from recently invaded sites (<10 years), to sites where toads have been present for >80 years (Fig. 1). Figure 2 shows examples of sites where the activity times of toads were primarily diurnal (a), both diurnal and nocturnal (b) and exclusively nocturnal (c). Toads videoed by day were oriented towards the baits, consistent with feeding behaviour (as also inferred by^6^; see Fig. 3).

#### Hypothesis 1. Toads are diurnal when cool moist conditions are available by day

**Figure 4:**
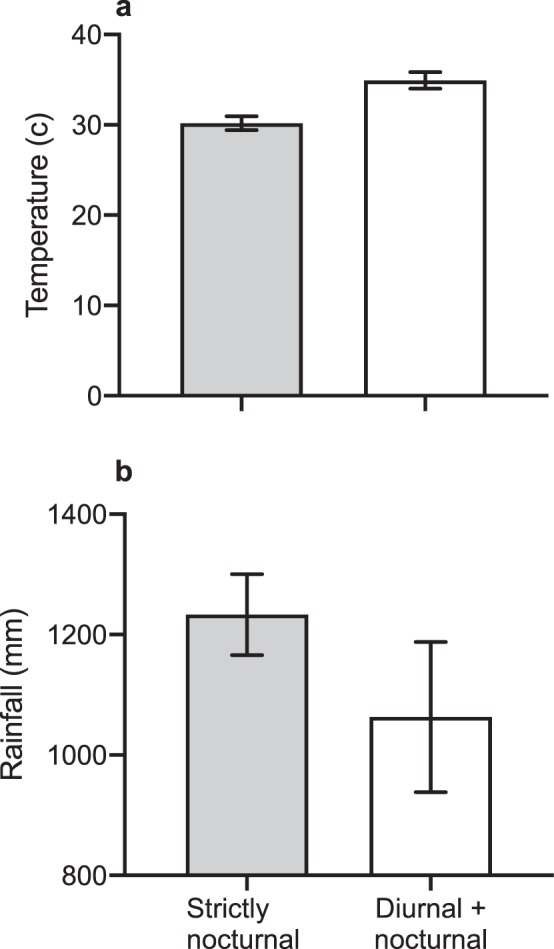
The (**a**) mean (± se) maximum temperature and (**b**) mean (± se) total rainfall during peak seasonal activity (Oct-May) for cane toads (*Rhinella marina*) at 34 sites across Australia where toads displayed strictly nocturnal activity, or diurnal and nocturnal activity.
- Weather conditions at the times of day that we recorded diurnal activity in cane toads were hotter and drier than when we recorded nocturnal activity in toads at the same sites (Oombulgurri temperature *F*_(1,536.4)_ = 291.26, *P* < 0.0001, Fig. 5a; Townsville temperature *F*_(1,112.5)_ = 161.65, *P* < 0.0001, Fig. 5b; Oombulgurri relative humidity *F*_(1,538.6)_ = 227.34, *P* < 0.0001 Fig. 5c; Townsville relative humidity *F*_(1,110.9)_ = 118.82, *P* < 0.0001 Fig. 5d).

Our analyses falsify predictions from this hypothesis, as follows:

- The sites where we detected diurnally active cane toads were hotter (Wilcoxon χ^2^_1_ 7.28* P* = 0.007; Fig. 4a) but received similar rainfall (Wilcoxon χ^2^_1_ 0.17 *P* = 0.68; Fig. 4b) as sites where we recorded only nocturnal activity.

**Figure 5:**
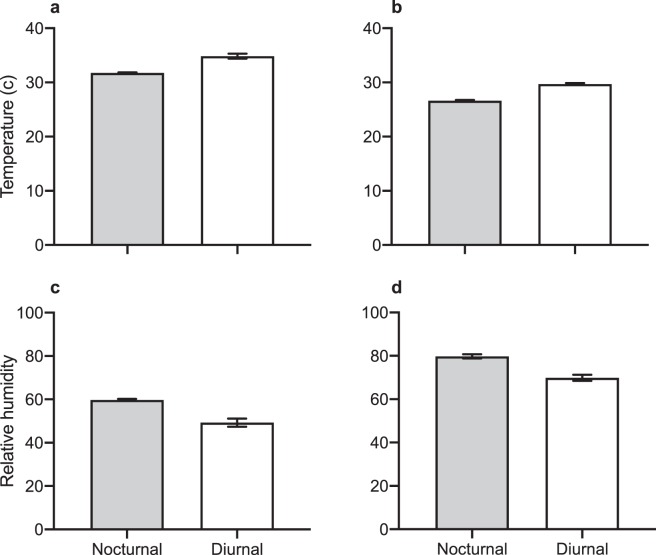
The mean (± se) temperature and humidity conditions during times of day when cane toads were active nocturnally and diurnally, at two sites where both of these activity patterns were common. Left-hand panels (**a**,**c**) show data for Oombulgurri (10.9% of toad activity occurred by day) and right-hand panels (**b**,**d**) for Townsville Town Common (29.3% of toad activity by day).

#### Hypothesis 2. Toads are diurnal when nocturnal temperatures are too low for activity

The higher frequency of diurnal activity in hotter sites (above) is incompatible with this hypothesis.

#### Hypothesis 3. Toads are diurnal when food is scarce

**Figure 6:**
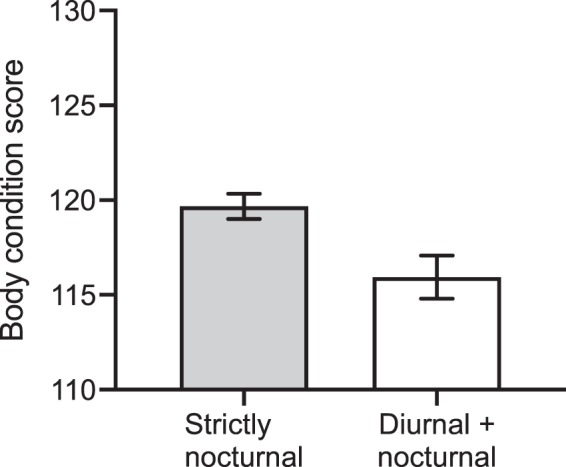
The mean (± se) body condition scores of cane toads (*Rhinella marina*) from populations where we recorded only nocturnal behaviour, and from populations where we recorded diurnal as well as nocturnal behaviour.
- the trend for the proportion of diurnal toads to increase with population density (Spearman ρ 0.35 *P* = 0.04; Fig. 7). Diurnal activity was not detected in the 15 sites where toads were least common.

The trend for diurnality in hotter climates (where maintenance metabolic requirements are higher) is consistent with this hypothesis. This hypothesis is further supported by:

- the trend for toads from populations that exhibited diurnal and nocturnal activity to be in poorer body condition compared to toads from populations that were exclusively nocturnal (ANOVA *F*_(1,928)_ = 5.15, *P* = 0.0235; Fig. 6).

**Figure 7:**
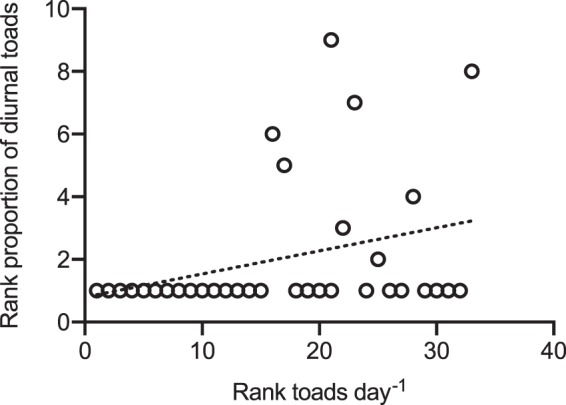
The relationship between the ranked abundance of cane toads (*Rhinella marina*) among the 34 populations that we surveyed (based on daily numbers of toads detected during active surveys as well as camera traps) compared to the incidence of diurnal activity (ranked proportion of toad diurnal activity).

### Radiotelemetry in French Guiana

We detected diurnal activity in 11% of radio-tracked toads (4 of 34) across three of our four sites (two beach sites and one rainforest site). All diurnal toads were males (but note that 23 out of the 34 tracked toads were males). Three individuals were active on only one of the five survey days, whereas one toad (at a beach site) was diurnally active on all five days. On the beach sites, diurnally active toads were actively hunting in open habitat (beach sand partially covered with vines) and exposed to solar radiation; the toad that was active in the rainforest was moving across the litter, protected from the sun by the canopy. Contrary to our expectations, active toads were more difficult to locate during the day than by night without telemetry, because their eye shine makes them readily detectable at night. This may lead to diurnal activity being underestimated by casual observers. Toads that were active at least once during the day were also active on each of the three tracking nights, and were thus also more active nocturnally than non-diurnal toads (which were active 83% of the nights), although this difference was not significant (estimate = 14.61 ± 9.66; t = 1.51; DF = 29; p = 0.14). Toads that were diurnally active were no different from the other toads in terms of mean body size (SUL; ANOVA on log data *F*_(1,29)_ =1.11 *P* = 0.30) or body condition (ANOVA on reciprocal data *F*_(1,29)_ =0.26, *P* = 0.62).

## Discussion

The extensive dataset provided by Doody *et al*.^6^ convincingly demonstrates that cane toads flexibly adjust their diel activity regimes based upon abiotic conditions, adding to an emerging consensus that behavioural flexibility is a significant contributor to invasion success in this species (e.g.^9,10^, as well as in other invasive taxa^11,12^). However, our more extensive spatial sampling shows that diurnal behaviour is present in a wide variety of habitat types both within the species’ native range and across its broad invasive spread in Australia, and is therefore not limited to areas where unique geological features moderate diurnal conditions. Doody *et al*.’s^6^ inference that diurnality is very rare in cane toads across most of their range was based upon general statements in published literature^6^, whereas empirical data from camera-trapping and active-searching tell a different story. Diurnal activity may be absent entirely from many populations (as seems to be the case in temperate-zone Australia); but in some populations, right across the species’ range, it is not unusual to find individuals active by day.

Given that adult cane toads are generally regarded as nocturnal, these records of toads active by day are surprising. Doody *et al*.^6^ suggested that *“Cane toads in Australia are nocturnal, probably because diurnal activity would subject them to intolerably hot and dry conditions in the tropical savannah during the dry season”*. That statement accords well with our own intuition, but not with the expanded dataset. Notably, the sites where we recorded extensive diurnal activity included some of the hottest and driest sites that we studied (Fig. 3; and see also^13^), such that diurnal activity occurred at these sites in markedly hot, dry conditions (Fig. 4). In the native range, diurnal activity occurred in open habitats (where the temperature of the surrounding sand could exceed 50.5 °C), as well as under the cover of the rainforest canopy.

Thus, we can dismiss the hypothesis that diurnal activity in cane toads is solely a response to favourable ambient conditions by day. Many sites along our eastern transect provide relatively cool moist conditions; and yet toads do not respond by becoming diurnal. Dense rainforest also provides relatively cool moist conditions during the day, but both the literature and our observations show that toads in these habitats remain mostly nocturnal. Likewise, our data militate against the idea that toads become diurnal to compensate for a lack of thermally suitable nocturnal foraging opportunities. Instead, diurnal foraging appears to be a response to food scarcity. Diurnal activity was more common in hotter climates, in high-density populations, and in sites where toads were in relatively poor condition. We suggest that high ambient temperatures in tropical sites elevate basal metabolic rates, requiring higher rates of food intake^14^; and high conspecific densities exacerbate competition for food, a major influence on feeding rates for cane toads^15,16^. Food-limitation thus may induce a shift in activity times, as has been reported in many other species^17,18^.

There may be multiple reasons why some toads are active by day. Sometimes they may need to move to avoid lethal conditions: for example, away from dangerously hot and dry diurnal shelters to nearby waterbodies^13^, or if amoebic dysentery impairs their water-retention ability^19^. More commonly, however, diurnal activity may allow toads to feed over a longer period, or on different types of prey, than would be possible under a strictly nocturnal regime. The videos from our camera traps (e.g., Fig. 3) strongly suggest that diurnal activity in these toads was at least partly driven by feeding opportunities (see also^6^).

Although adult cane toads are primarily nocturnal, a shift towards diurnal activity may not be as challenging for this species as for many other obligately nocturnal taxa. First, the phylogenetic lineage that includes cane toads also includes taxa that are diurnally active either usually (e.g., *Rhinella hoogmoedi*^20^) or facultatively (*Melanophryniscus cambaraensis*^21^). Secondly, as in many toad species, cane toads are diurnal during the metamorphic life stage; this adaptation is thought to facilitate rapid growth during the juvenile stage^22^, and can also reduce the risk of cannibalism^23^. Thus, the basic bauplan of a toad allows it to be active by day as well as by night. The abiotic challenges of heat and desiccation are partially reduced by the large body size of adult cane toads, that confers resistance against rapid changes in temperature or hydration state^24^. Lastly, rapid evolution of behavioural and physiological traits within the toad invasion of Australia suggests that these animals can swiftly adapt to challenges encountered during range expansion^25–28^.

A geographic shift in the frequency of diurnal behaviour in cane toads may have important consequences for the spread and impact of this ecologically-damaging pest species^29^. First, the ability to flexibly adjust times of activity may enable toads to colonise sites where nocturnal conditions are unsuitable – thus expanding the range of the invasion. Second, toads that are active by day may feed upon a different array of prey taxa, modifying their impact. Third, diurnal activity may bring the toxic toads into direct contact with diurnally foraging native predators that might thereby experience earlier or more severe consequences from interacting with toads. Behavioural flexibility in the diel timing of activity – a trait inherited from the native range – may thus affect both the spread and the impact of this iconic invasive species.

## Data Availability

Data will be provided upon reasonable request to the corresponding author.
